# Design, Fabrication, and Experimental Validation of Microfluidic Devices for the Investigation of Pore-Scale Phenomena in Underground Gas Storage Systems

**DOI:** 10.3390/mi14020308

**Published:** 2023-01-25

**Authors:** Alice Massimiani, Filippo Panini, Simone Luigi Marasso, Nicolò Vasile, Marzia Quaglio, Christian Coti, Donatella Barbieri, Francesca Verga, Candido Fabrizio Pirri, Dario Viberti

**Affiliations:** 1 Politecnico di Torino, 10129 Torino, Italy; 2 CNR-IMEM, 43124 Parma, Italy; 3 Stogit-Snam, 26013 Crema, Italy; 4 Istituto Italiano di Tecnologia, 16163 Genova, Italy

**Keywords:** microfluidics, underground gas storage, pore-scale

## Abstract

The understanding of multiphase flow phenomena occurring in porous media at the pore scale is fundamental in a significant number of fields, from life science to geo and environmental engineering. However, because of the optical opacity and the geometrical complexity of natural porous media, detailed visual characterization is not possible or is limited and requires powerful and expensive imaging techniques. As a consequence, the understanding of micro-scale behavior is based on the interpretation of macro-scale parameters and indirect measurements. Microfluidic devices are transparent and synthetic tools that reproduce the porous network on a 2D plane, enabling the direct visualization of the fluid dynamics. Moreover, microfluidic patterns (also called micromodels) can be specifically designed according to research interests by tuning their geometrical features and surface properties. In this work we design, fabricate and test two different micromodels for the visualization and analysis of the gas-brine fluid flow, occurring during gas injection and withdrawal in underground storage systems. In particular, we compare two different designs: a regular grid and a real rock-like pattern reconstructed from a thin section of a sample of Hostun rock. We characterize the two media in terms of porosity, tortuosity and pore size distribution using the A* algorithm and CFD simulation. We fabricate PDMS-glass devices via soft lithography, and we perform preliminary air-water displacement tests at different capillary numbers to observe the impact of the design on the fluid dynamics. This preliminary work serves as a validation of design and fabrication procedures and opens the way to further investigations.

## 1. Introduction

The urgent global need to decarbonize the energy sector and strengthen energy security is pushing toward the development and improvement of new methods for energy production and storage. In this framework, underground gas storage facilities are recognized as one of the best large-scale storage options since geological formations such as depleted hydrocarbon reservoirs and deep aquifers offer large storage capacity and safety [1]. Underground natural gas storage started in 1915 and is currently a reality, with nearly 700 facilities developed worldwide. CO_2_ storage is widely acknowledged to be an essential step toward decarbonization: 30 large-scale facilities are operated worldwide and over 160 are underway [2,3,4]. Eventually, underground hydrogen storage is more and more regarded as an opportunity—if not a need—to fully exploit the potential of renewable energy [5,6,7,8]. The main issues related to an underground storage system are capacity (the volume of fluids that can be contained by the system), injectivity (the amount of fluid that can be injected into the system over a period of time), integrity (the system’s ability to retain injected fluids without significant losses over the long term), and safety (mainly, absence of induced seismicity, fault (re) activation and ground movements). To address these issues significant fundamental and experimental research is being undertaken at different scales and in different disciplines.

To gain a better understanding and achieve improved modeling ability of the fluid-dynamics aspects related to the conversion of depleted gas reservoirs to hydrogen storage, it is fundamental to investigate the pore-scale phenomena that occur in the reservoir. Rock properties such as grain size, pore size distribution, porosity and absolute permeability, and rock-fluid interaction properties such as wettability and capillary pressure affect pore-scale phenomena and thus both storage capacity and injectivity. Furthermore, viscosity, density, and interfacial tension of the fluids that coexist in the pore space play a fundamental role in flow dynamics.

Transparent microfluidic devices have found application in the oil and gas industry because they can be designed to reproduce porous media networks, allowing for the direct visualization of the fluid dynamics occurring at the pore scale. This characteristic of microfluidics solves one of the main drawbacks of traditional core-flooding experiments, which require powerful imaging techniques to provide insights into pore-scale dynamics [9]. Moreover, rock samples are usually not reusable after an experiment. Another advantage of microfluidic experiments is that the rock properties, such as porosity, permeability, pore size distribution and wettability, can be manipulated, allowing for the fabrication of a wide range of samples.

In this work, we have designed, characterized, and fabricated two different microfluidic devices, and performed preliminary multiphase flow tests using air and water. The first designed geometry is a regular pattern, while the second geometry is extracted from a thin section of a Hostun rock sample. The two designed patterns differ in terms of tortuosity, heterogeneity, and regularity of the porous volume. The path-finding algorithm A* [10] and CFD simulation have been used to characterize the pore space of the designed patterns. Soft lithography has been chosen to fabricate PDMS-glass devices. In particular, the aim of this work is to further understand how different designs affect the fluid flow dynamics and to better comprehend which kind of information can be extracted from different patterns.

## 2. Theoretical Background

In order to investigate the physical phenomena that occur in microfluidic devices, it is necessary to introduce a few parameters that play an important role in multiphase flow in porous media. In microfluidic experiments, we observe multiphase flow behavior directly in the pore space, at a microscopic scale of investigation, which is characterized by a set of parameters that, in turn, have an influence on macroscopic parameters such as porosity and permeability. Details on the theory of pore-scale phenomena and pore-scale modeling can be found in [11,12]. Multiphase flow parameters considered in this work are capillary number, viscosity ratio, and capillary pressure [13]. In fluid dynamics in porous media, the fluid velocity is usually very small and therefore the fluid flow is laminar (Re<1); this flow condition is satisfied in microfluidic experiments as well.

The capillary number Ca is a dimensionless number that represents the ratio of viscous force to the capillary force and is calculated as follows [12,13]:

(1) $$Ca=\frac{\mu U}{\sigma },$$

where μ is the dynamic viscosity, σ is the interfacial tension and U is the characteristic velocity which in this work is the interstitial velocity, defined as Darcy’s velocity divided by porosity. When Ca>1, viscous force dominates the fluid flow whereas the capillary force dominates the fluid flow when Ca≪1. The viscosity ratio M is defined as the ratio between the viscosity of the displaced fluid to that of displacing fluid [13]:

(2) $$M=\frac{\mu_{displaced}}{\mu_{displacing}}.$$

The higher the viscosity ratio and the capillary number, the more stable the fluid displacement. When the capillary number decreases, capillary forces start to prevail over the viscous forces, pore size distribution controls the invasion and capillary fingering may occur. When the mobility ratio decreases, viscous fingering occurs, and the front cannot be stabilized [14]. Typically, when M≪1, viscous fingering is likely to occur, which leads to a low occupant efficiency of the displaced fluid. When M>10, capillary fingering or stable displacement can occur. Capillary pressure has an inherent pore-scale nature and is calculated as the pressure difference across the curved interface between two immiscible fluids in equilibrium [15]:

(3) $$P_{c}=P_{nw}-P_{w},$$

where $P_{nw}$ is the pressure of the non-wetting phase and $P_{w}$ is the pressure of the wetting phase. The relation between the capillary pressure and the curvature of the interface is given by the Young–Laplace equation [9]:

(4) $$P_{c}=P_{nw}-P_{w}=\sigma \kappa ,$$

where κ is the curvature of the interface. If considering a capillary tube, the equation can be simplified as [15]:

(5) $$P_{c}=\frac{2\sigma \;cos\theta }{r},$$

where θ is the contact angle, and r is the radius of the capillary tube. The actual microscale geometry of a porous matrix is however too complex, and it is not possible to account for all the fine details such as the local values of pore width. Instead, macroscopic models, known as Darcy or continuum scales are used, which average the capillary pressure values over a Representative Elementary Volume (REV), considering a large number of pores. The non-wetting and the wetting phase pressures become the “average” equivalent capillary pressure, and they are all related to the macroscopic saturation of the two phases as [15]:

(6) $$P_{c}\left(S^{w}\right)=P^{macro}{}_{nw}-P^{macro}{}_{w}.$$

**Figure 1:**
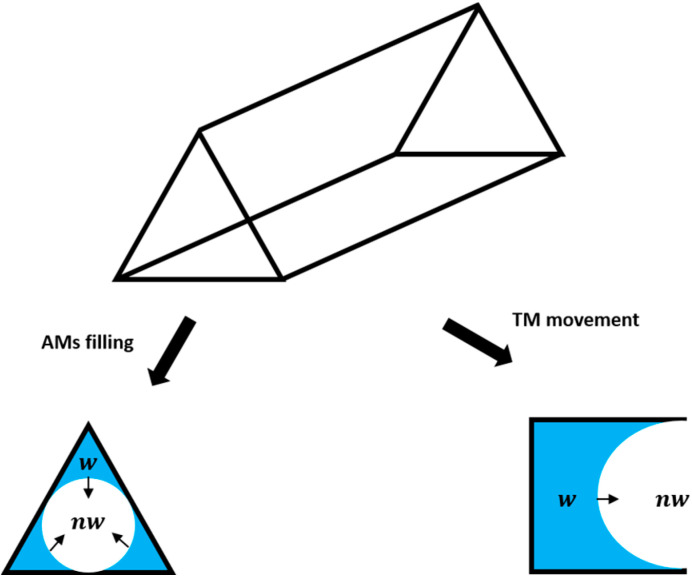
Arc and Terminal Menisci with respect to the three axes; *w* denotes the wetting phase and *nw* the non-wetting phase [16].

When considering a dynamic condition, this is no longer valid, and the fluids enter the so-called non-equilibrium regime. The fluid pressures vary with time and space and the viscous forces gain importance. At the microscopic level, all local phenomena are governed by the local capillary pressure, and by the local capillary pressure instabilities. The displacement sequence on a pore-by-pore basis is composed of a sequence of equilibrium steps, where the fluids reach the equilibrium position (energy balance) following the Young–Laplace equation. For slow displacement, this also determines the threshold capillary pressure at which one phase is able to displace the other. The threshold capillary pressure depends on the contact angles of the fluids, and at the macro-scale, it determines the way each phase flows and how much is displaced [11]. In general, according to the Young–Laplace equation, the non-wetting phase has sufficient pressure to enter the wider pores first, while the wetting phase preferentially fills the narrower channels. From a macroscopic point of view, two different processes exist: drainage and imbibition. During drainage, the overall saturation of the non-wetting phase increases. During imbibition the overall saturation of the wetting phase increases. However, at the pore scale, fluid dynamics are more complex, and the displacement front may be locally disturbed and interrupted. An important aspect to consider is that porous networks have very irregular cross-sections. The wetting phase (water), which is displaced by the non-wetting phase (oil) during the first drainage, adheres to the rock surface and remains in the crevices and corners in the form of interconnected wetting layers, able to swell and move while water is newly injected during imbibition. This can be modeled with a triangular- or square-shaped cross-section as shown in Figure 1 [16]. This condition induces two specific configurations of the moving fluid interface, namely Arc Menisci (AM) and Terminal Menisci (TM). In the first case (AM), the wetting phase does not occupy the center of pores and throats, while the opposite occurs in the latter case (TM). In Figure 1, both AM and TM are shown to better understand the dynamics of fluid filling according to the two configurations. The dynamics of an interface between two immiscible fluids in a porous media is characterized by spontaneous reconfigurations due to local instabilities that lead to irreversible jumps or re-arrangements of the menisci [17]. These pressure instabilities may cause the abruption of the fluid front and induce trapping phenomena. Overall, both the local pressures and the local pressure instabilities also determine the way fluids percolate through the medium.

## 3. Design of Microfluidic Devices

The micromodels used in this work are designed to observe the multiphase flow and pore-scale phenomena. In this Section, we describe the procedure used for extracting characteristic parameters such as porosity, tortuosity, and average and smallest pore size of the micromodels. The values obtained for the considered geometries are all summarized in Table 1. These parameters are useful because they represent rock properties that influence flow behavior and by varying them, it is possible to obtain micromodels able to mimic different reservoir rock types.

**Figure 2:**
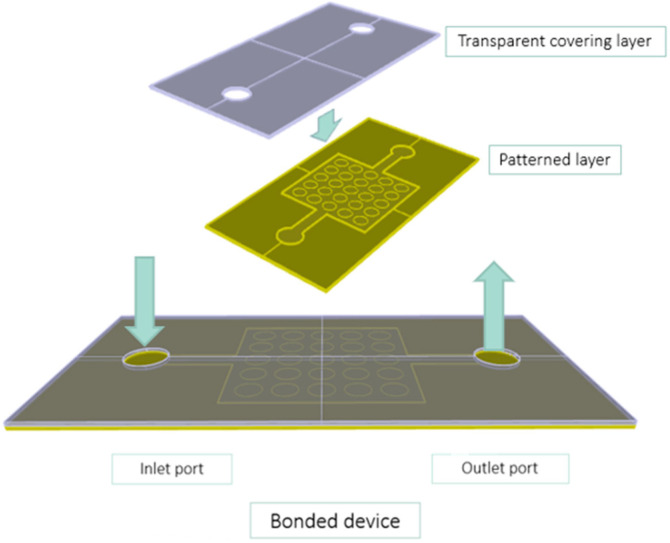
Sandwich structure of a microfluidic device, showing the two bonded layers. The patterned layer reproduces the designed geometrical features. The covering layer confines the fluid, typically hosts the inlet and outlet ports and allows for the visualization of the pore-scale phenomena. Inlet and outlet ports can also be carved out of the patterned layer.

As shown in Figure 2, a microfluidic chip is typically constituted by two bonded layers: the patterned slab, which hosts the microfluidic circuit, and the transparent covering layer, which seals the circuit and allows for the visualization of the fluids inside. For microfluidic devices representative of rocks, the microfluidic patterned core mimics the porous network, and the inlet and outlet channels connect the pore network to the inlet and outlet ports. The internal features range from a few to hundreds of micrometers. The inlet and outlet system allows for the fluids to be injected and extracted.

**Figure 3:**
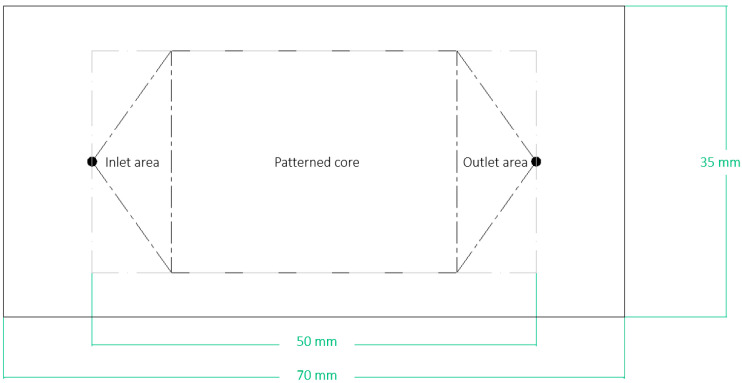
Layout of the microfluidic device, showing the inlet and outlet are and the pattern. The linear dimensions refer to the total extension of the devices and the inlet–outlet port distance.

In our devices, the total dimensions are 70 mm × 35 mm, and the distance between the inlet and the outlet port is 50 mm (Figure 3).

**Figure 4:**
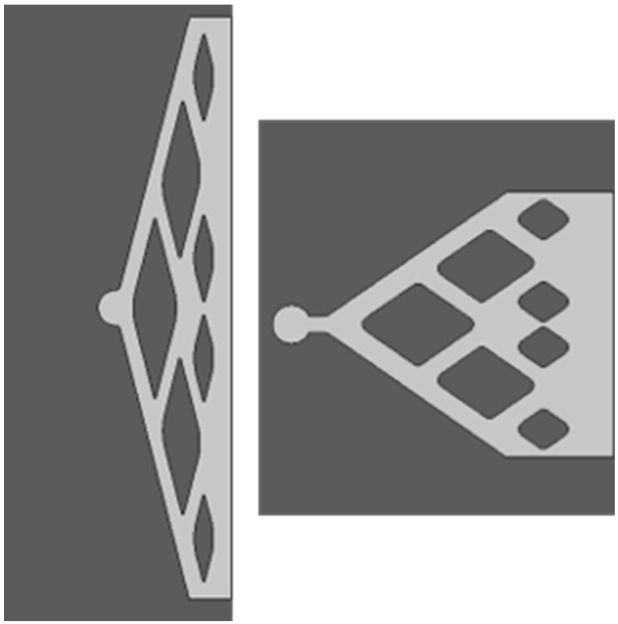
Design of the inlet geometries.

Inlet areas have been designed to obtain a flat fluid front at the entrance of the patterned core. The branched layouts shown in Figure 4 were validated by CFD simulation and it was verified that the velocity profile was almost constant at the pattern entrance, to avoid favorable fluid percolation paths. They also have been scaled to become adapted to different dimensions. The same design was applied to the outlet area.

For the development of the micromodel patterns, two different solutions are proposed: a regular grid and a real-rock image mosaic. These two patterns offer an incremental geometrical complexity and different degrees of control over the geometrical properties. Dimensions are kept inside the constraints of fabrication feasibility, meaning that no proposed feature is smaller than a few microns.

### Regular Grid and Real Rock Image

**Figure 5:**
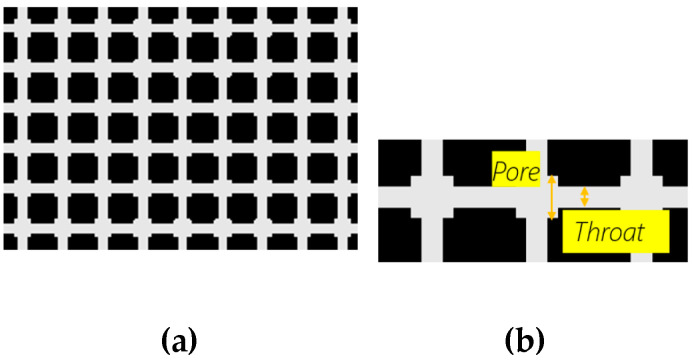
GRID01, constituted by squared pores, and a pore-throat to pore-body ratio of 2:1. The total number of pores is 64000. (**a**). Throats are 25 µm, and pores are 50 µm wide (**b**).

**Figure 6:**
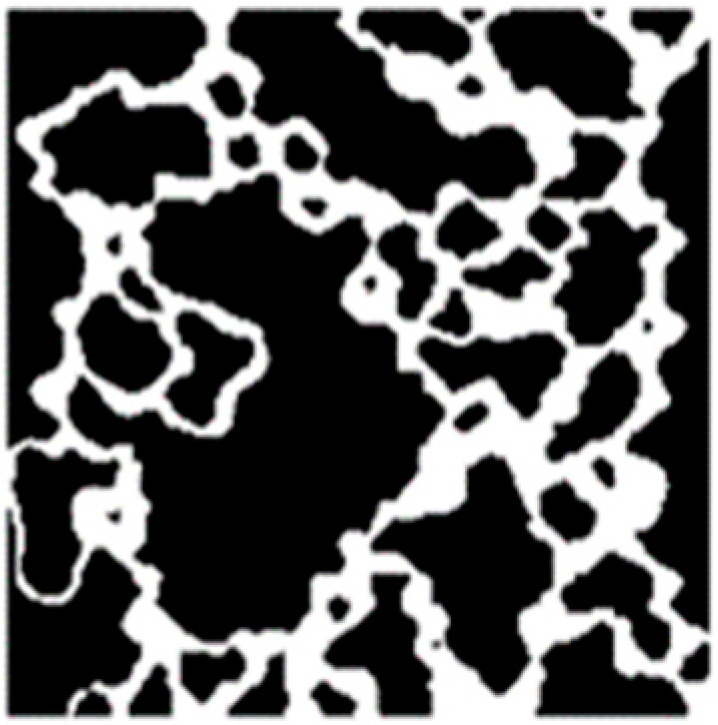
HO49Y. Hostun sand is relatively homogeneous, characterized by well-sorted predominantly quartz angular grains.

Two different micromodels were designed and tested: a regular grid, named GRID01, and a mosaic of a real rock image, named HO49Y. The regular grid is a regular pattern because all throats and all pores have the same dimensions (Figure 5). The grid has throats of 25 µm and squared pores 50 µm wide. Tortuosity is 1, but the 2:1 pore-throat ratio favors local pressure instabilities and therefore the occurrence of pore-scale phenomena, such as snap-off. The square shape of the pores was designed to create corners in which the wetting layers may reside more easily [11]. To replicate the 2D geometrical complexity of real rocks, 2D images of thin sections of rocks have been considered. In particular, an image was obtained from a thin section of a 3D sample of the Hostun rock (Figure 6). Hostun sand has *D*_50_ = 300 μm. First, preliminary image processing was applied to convert grayscale images to binary images. The microfluidic layout was created by removing the dead ends. In HO49Y, tortuosity is equal to 1.34. The borders of the images were then slightly modified to make it possible to replicate the images as a mosaic without interrupting the fluid flow. This is a common procedure, as reported in the literature [18]. The mosaic is needed because of the small dimensions of the images, obtained from microscopic imaging.

Both micromodels have been fully characterized by extracting data with dedicated procedures. In particular, the values of tortuosity were calculated via CFD simulation, and the static values of average pore size and smallest pore size using a procedure based on the A* algorithm [10,19,20]. All properties were extracted from a Representative Elementary Volume (REV). The results are all incorporated into Table 1.

## 4. Device Fabrication

**Figure 7:**
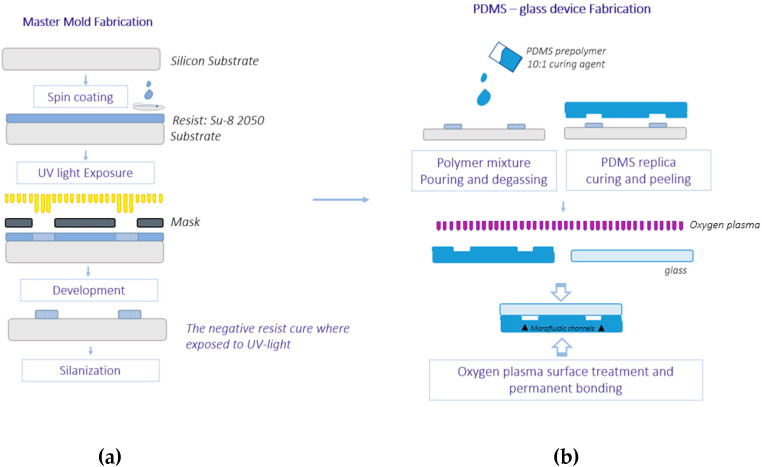
Scheme of the soft lithography process used for the fabrication of the microfluidic devices. (**a**) Fabrication of the master mold via lithography (**b**) PDMS casting and molding, followed by Oxygen plasma bonding.

The fabrication of our PDMS microdevices is based on soft lithography (Figure 7). A first lithographic step is required to fabricate the master molds, on which the PDMS is cast to create the microfluidic structure. All molds consist of a silicon wafer on which a polymeric (photoresist) structure is built. The PDMS replica will present channels in correspondence of the photoresist structure. The devices are sealed with borosilicate glass slides. The fabrication has been fully carried out in our lab, following the steps below:

- Glass–chrome masks for each pattern are prepared via laser writing. The chrome–glass mask blanks are purchased by MB Whitaker & Associates. A LaserWriter system by MICROTECH is used to transfer the desired pattern onto the blanks and create the final mask. The masks undergo a process of resist development, chrome etching and piranha cleaning to be ready to use. Optical inspection is performed to guarantee that the final mask has no dimensional mismatch with respect to the designed geometry.
- Su-8 2050 is used as a photoresist for the fabrication of the master molds. The Su-8 2050 belongs to a series of negative photoresists. The resist is spin-coated on a silicon wafer and soft-baked. The sample is then loaded inside a Mask Aligner and exposed to the UV-light beam. A post-exposure bake follows, and the resist is then developed with appropriate solvents to remove the undesired material and keep the microfluidic pattern mold only. A final hard bake is added to ensure that SU-8 properties do not change in actual use (Su-8 2000 Processing guidelines). All baking steps are necessary to optimally cure the photoresist and process the structures. A level hotplate with good thermal control and uniformity is required. Two steps at 65 °C and 95 °C are performed during soft bake and post-exposure bake. The hard bake reaches 150 °C.
- The sample undergoes wet silanization after the hard bake. This treatment makes the silicon surface more hydrophobic, which means that PDMS will have less affinity to it and will be easier to detach. Toluene and Trichloromethylsilane (10.1 ratio) are used for the silanization bath.
- PDMS Sylgard 184 is used for the microfluidic replica. A mixture of the prepolymer and the curing agent in a 10:1 ratio is poured on the master mold after degassing and let cure.
- The replica is then peeled off the mold. The inlet and the outlet holes are drilled with a puncher, to achieve a hole diameter of 1 mm.
- To seal the device, both the PDMS replica and the glass slides are exposed to an oxygen plasma surface treatment before being permanently bonded together by the application of a gentle pressure. Oxygen plasma treatment guarantees irreversible bonding and good sealing resistance even after multiple cycles of experiments [21].

**Figure 8:**
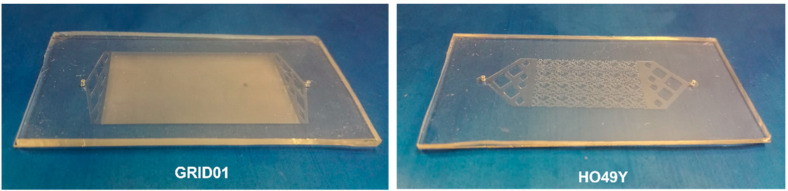
PDMS-glass microfluidic devices.

**Figure 9:**
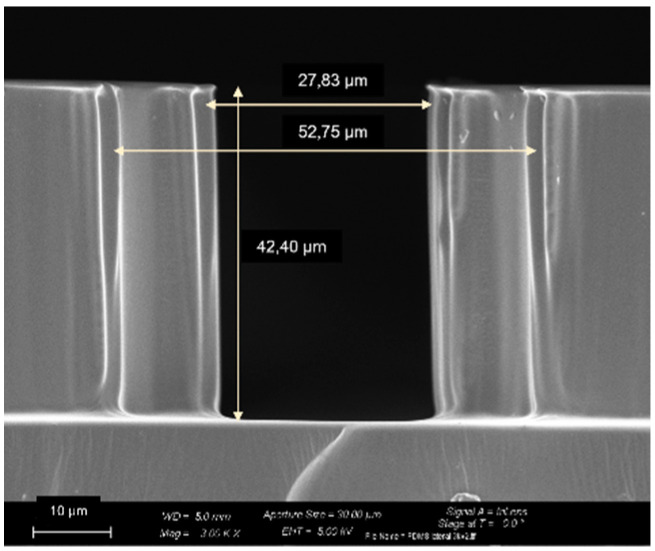
**A** FESEM image of a cross section of the PDMS replica hosting the regular grid.

Figure 8 shows the final PDMS-glass devices. The PDMS replicas have been characterized and the quality was checked via optical inspection, prior to oxygen bonding. The measurement performed with the optical microscope shows the good reproducibility of the pattern details and dimensions throughout the fabrication process, if compared to the CAD design. The depth of the channels has been measured with a contact profilometer and double-checked via FESEM imaging on the regular grid (Figure 9). The averaged measured depth resulted to be 41.2 µm for the regular grid, and 42.9 µm for the Hostun pattern.

### Experimental Set-Up and Fluid-Flow Tests

**Figure 10:**
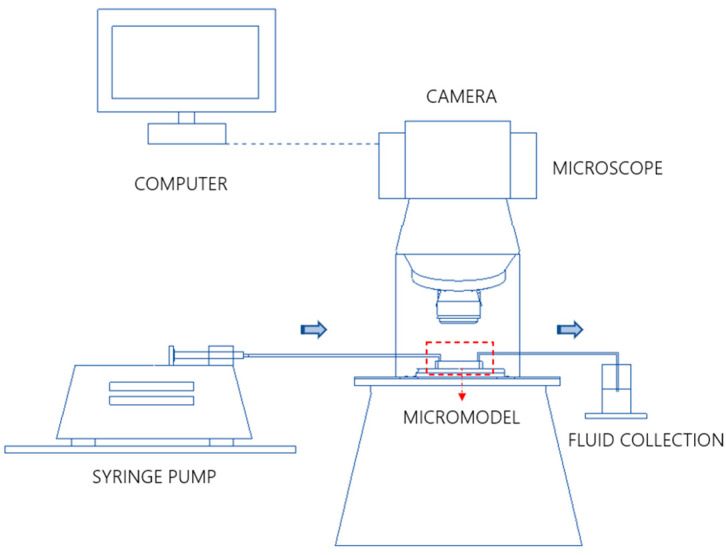
Experimental setup.

Figure 10 shows the experimental setup for the fluid flow preliminary tests. The microfluidic chip is placed under a DSX1000 Olympus Microscope. The inlet and outlet ports of the device are connected to a system of tubes, for the injection and the extraction of fluids. A Harvard syringe pump is used to inject the fluids using Hamilton gas tight syringes. A container is positioned at the end of the outlet tube to collect the ejected fluids. In this work, we performed drainage and imbibition experiments on the micromodels GRID01 and HO49Y at different flow rates in order to vary the capillary number and investigate different flow regimes. The fluids used are air and water, where air is the non-wetting phase and water the wetting phase. The interfacial tension between the two fluids is 0.072 N/m. The viscosity of the water and the air are respectively 1 cP and 0.01 cP. We run the experiments at capillary numbers from $10^{-6}$ to $10^{-3}$. The flow dynamics is recorded with the microscope camera. The contrast of the pictures is increased in ImageJ (NIH) to better visualize the two fluids.

## 5. Results and Discussion

In this Section, we discuss the observations and results of the experiments carried out on microfluidic devices. In particular, the possibility to observe the whole area of the devices allowed us to monitor the device filling and visualize the flow patterns, which are both influenced by the capillary number and viscosity ratio. At higher magnifications, we could also capture local pore-scale phenomena.

### 5.1. Discussion on Dimensionless Numbers and Flow Conditions

Capillary number and viscosity ratio are expected to play a substantial role in the displacement pattern and displacement efficiency during the multiphase fluid flow in porous media. Under the influence of these two dimensionless numbers, the fluid flow pattern of the injected fluid can be categorized as either viscous fingering, capillary fingering or stable displacement. In our experiments, the viscosity ratio is equal to -2 for drainage and 2 for imbibition.

**Figure 11:**
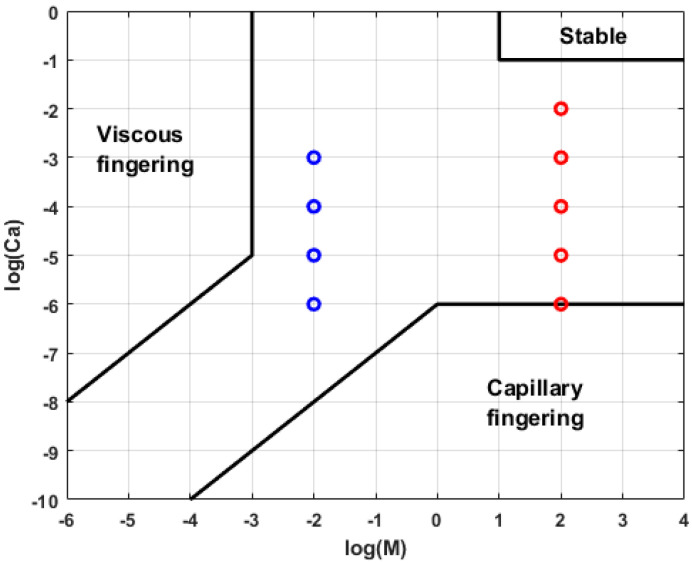
logCa–logM phase diagram. Blue points refer to the drainage experiment conditions and the red points to the imbibition experiment conditions.

Usually, in order to identify the flow regime occurring in the micromodels, the logCa-logM phase diagram proposed by Lenormand et al. [13] is used. In Figure 11 we show the location of our experiments in the logCa–logM diagram. Our experiments are located within the transition area between capillary fingering and viscous fingering on the Lenormand diagram. The boundaries of the logCa–logM phase diagram are system dependent [22] and therefore the boundaries reported in Lenormand et al. [13] are not necessarily applicable to our micromodel. The establishment of the connected air phase, stopped by narrow pore throats, may show the importance of capillary fingering at low/medium Ca.

**Figure 12:**
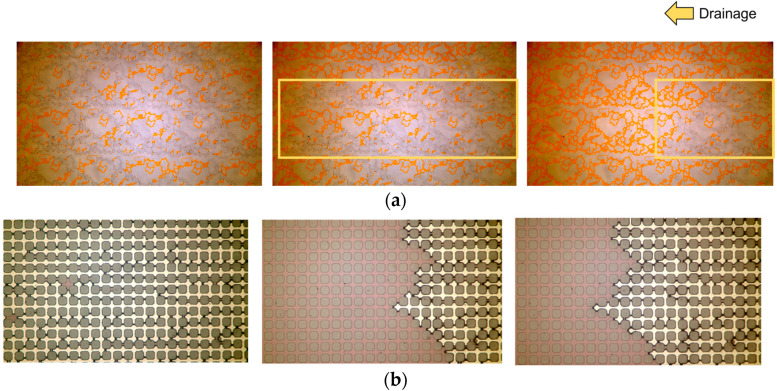
Images of the drainage process at $Ca=10^{-4}$ and at three different times for (**a**) HO49Y and (**b**) GRID01 grid. In the last time-step, the flow reached steady-state conditions. Air is white and water is orange in (**a**) and pink in (**b**).

**Figure 13:**
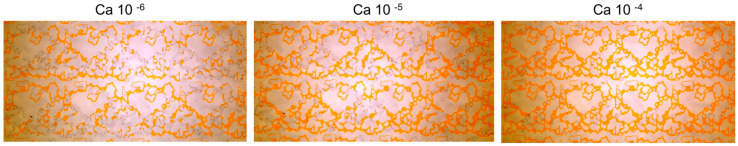
Images of the final saturation induced by imbibition processes at three different capillary numbers in HO49Y.

First of all, we run drainage tests. In this case, the system is initially fully saturated with water and then air is injected at different flow rates. We could observe a very uniform fluid front at the inlet of the devices, which validates the design of the inlet channels. After steady state conditions are reached, i.e., there is no variation of flow patterns in time, the test is stopped, and the final configuration and saturation are used as the initial configuration for imbibition experiments. In Figure 12, the evolution of the air front is shown at different times at a capillary number equal to $10^{-4}$ for HO49Y and GRID01. When air enters the HO49Y micromodel, two main flow paths are exhibited at the center of the image, indicating viscous fingering. A viscous fingering behavior was also observed in GRID01, although at the magnification used, it cannot be appreciated in the images. The final configuration reached at the end of the drainage experiment is the starting point for the imbibition test. In Figure 13, we show the steady state conditions at the end of the imbibition test at three different capillary numbers. It is evident that by increasing the capillary number, the displacement efficiency increases and the residual air remaining in the porous medium decreases.

### 5.2. Discussion on Observed Pore-Scale Phenomena

**Figure 14:**
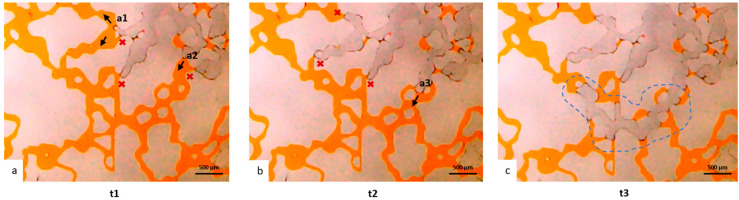
Evolution of the gas front during the drainage process in HO49Y at time t1 (**a**), t2 (**b**) and t3 (**c**). From time t1 to time t3 two seconds elapsed.

**Figure 15:**
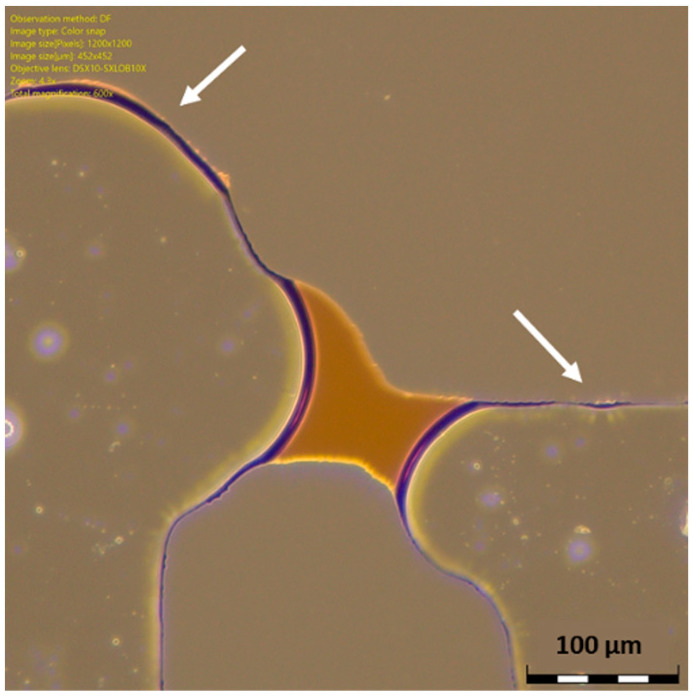
Microscope picture of the wetting layers observed in HO49Y.

In drainage processes, the non-wetting phase fills the largest pores while the wetting phase remains in the smaller pore throats because of the highest capillary pressure [11]. This can be clearly seen in HO49Y, as shown in Figure 14. For instance, given the configuration shown in the sequence of frames in Figure 14, it can be visualized how the gas fronts advance first from the positions having the wider channel width. In particular, in Figure 14a,b, we see the gas fronts preferring the paths indicated by the black arrows, as the channel width at the locations a1 and a2 is respectively 69 µm and 79 µm, while it is 34 to 58 µm in the channels indicated by the red crosses. In Figure 14b,c, it is visible that the gas prefers moving from location a3, which is 130 µm wide, while it does not move at the locations indicated by the red crosses, which are 34 to 64 µm wide. Moreover, after the gas overcomes smaller throats, we see a rapid filling of the subsequent bigger pores and throats, a phenomenon called “Haines Jumps” [11], which is particularly evident in the sequence of pores invaded in Figure 14c (dashed blue line). At higher magnification it was also possible to notice how the irregularity of the walls induced the formation of wetting layers, indicated by the white arrows in Figure 15. These layers play a role in pore-scale phenomena [11], as they may act as swelling arc menisci, but their influence must be further investigated.

**Figure 16:**
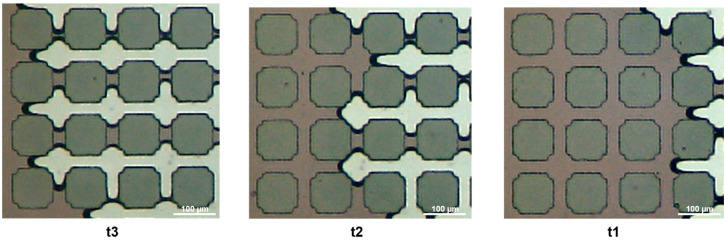
Evolution of the gas front during the drainage process on GRID01.

**Figure 17:**
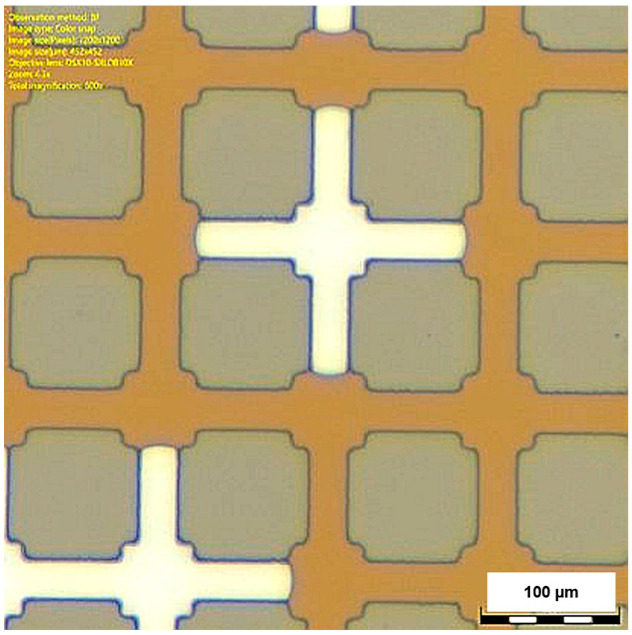
Microscope picture of GRID01. Wetting layers are not visible.

Differently, GRID01 does not present heterogeneity in the structure. The fluid front proceeds along the direction aligned with the fluid injection, and leaves trapped water in the perpendicular channels (Figure 16). The directionality of the flow and its impact on the residual trapping should be further investigated. Heines jumps cannot take place because of the regularity of the structure. However, the gas rapidly fills the pores and moves slower along the throats. Evidence of wetting layers was not found for the regular pattern, as seen in Figure 17; their presence should be further investigated during the imbibition processes.

### 5.3. Discussion on Methodologies and Future Perspectives

To serve our purposes of preliminary testing the imbibition and drainage processes in micromodels representative of reservoir rock formations, the combination of air and water provided a reliable observation of gas-brine fluid dynamics at the pore scale. Further investigation will be carried out using an exact combination of fluids because there are phenomena, such as dissolution, that may extensively vary according to the substances. In particular, the analysis of static and dynamic contact angles and contact angle hysteresis will be taken into account and discussed according to the fluids involved. Moreover, in order to reproduce the correct fluid behavior at reservoir conditions, reservoir pressure and temperature will be considered. Reservoir thermodynamic conditions change with depth and, when taken into account, can improve the representativeness of microfluidic experiments. Reasonable reservoir conditions can be estimated by calculating the pressure according to the hydrostatic gradient and the temperature according to the geothermal gradient. For example, at a reservoir depth of 3000 m, the static pressure is about 300 bar and the temperature is about 100 °C.

## 6. Conclusions

In the work presented in this paper, we designed and fabricated two microfluidic devices with the purpose of investigating pore-scale phenomena that occur in reservoir rocks during gas injection and withdrawal during underground gas storage operations. Multiphase flow experiments were performed on the two microfluidic devices and different pore-scale and trapping phenomena were observed. We could observe that the microfluidic device hosting the regular pattern would induce fewer pore-scale phenomena compared to the Hostun pattern, as expected. This is induced by the regularity of the structure and the very low tortuosity. On the other hand, the regular pattern allows to better isolate the effect of specific factors such as channels-flow alignment and pore-throat ratios. It would be interesting to modify the angles of the channels to increase tortuosity and increase the heterogeneity of the structure. The Hostun pattern allowed us to visualize more complex pore-scale phenomena, such as Heines jumps, and appears to be a promising platform for further analysis. The designed inlet channels demonstrated to serve the purpose of simulating homogeneous fluid front at the entrance of the porous pattern. New types of patterns will be investigated in future works.

## Figures and Tables

**Table 1:** Values of porosity, average pore size and smallest pore size of the micromodels.

Name	Porosity 2D	Average Pore Size (µm)	Smallest Pore Size (µm)
GRID01	0.34	-	25
HO49Y	0.32	96.52	21

## Data Availability

The data presented in this study are available in the article.
